# Anemia and chronic kidney disease are associated with poor outcomes in heart failure patients

**DOI:** 10.1186/1471-2369-7-3

**Published:** 2006-03-06

**Authors:** Jean-Christophe Luthi, W Dana Flanders, Michel Burnier, Bernard Burnand, William M McClellan

**Affiliations:** 1 Institute of Social and Preventive Medicine, University of Lausanne, Switzerland; 2 Health Observatory, Canton of Valais, Switzerland; 3 Epidemiology Department, Rollins School of Public Health, Emory University, Atlanta, USA; 4 Nephrology Department, CHUV, Lausanne, Switzerland; 5 Georgia Medical Care Foundation, Atlanta, USA

## Abstract

**Background:**

Chronic kidney disease (CKD) has been linked to higher heart failure (HF) risk. Anemia is a common consequence of CKD, and recent evidence suggests that anemia is a risk factor for HF. The purpose of this study was to examine among patients with HF, the association between CKD, anemia and inhospital mortality and early readmission.

**Methods:**

We performed a retrospective cohort study in two Swiss university hospitals. Subjects were selected based the presence of ICD-10 HF codes in 1999. We recorded demographic characteristics and risk factors for HF. CKD was defined as a serum creatinine ≥ 124 956;mol/L for women and ≥ 133 μmol/L for men. The main outcome measures were inhospital mortality and thirty-day readmissions.

**Results:**

Among 955 eligible patients hospitalized with heart failure, 23.0% had CKD. Twenty percent and 6.1% of individuals with and without CKD, respectively, died at the hospital (p < 0.0001). Overall, after adjustment for other patient factors, creatinine and hemoglobin were associated with an increased risk of death at the hospital, and hemoglobin was related to early readmission.

**Conclusion:**

Both CKD and anemia are frequent among older patients with heart failure and are predictors of adverse outcomes, independent of other known risk factors for heart failure.

## Background

Heart failure (HF) is a common and serious condition that affects more than four million people in the United States [1]. Approximately 400,000 new cases are diagnosed each year, with mortality 6 years after diagnosis of 80% in men and 65% in women [1]. In Europe, the prevalence of symptomatic heart failure in the general population is estimated to range from 0.4% to 2% [2]. In Switzerland, approximately 210,000 people have HF [3]. Chronic kidney disease (CKD) is also a major health problem resulting in considerably increased morbidity, mortality and in high costs [4]. Furthermore, in the last decade, the prevalence of both CKD[5,6], and HF has been rising steadily [7-9]. Anemia is a frequent complication of chronic kidney disease, primarily due to failure of erythropoietin production to respond to decreased haemoglobin concentration [10,11]. Anemia has also been found to be a risk factor for cardiovascular disease and in particular for HF [12,13]. In a study conducted in one Swiss university hospital the prevalence of anemia among heart failure patients was 15% [13].

Furthermore, several studies have also shown that anemia with the presence of heart failure was a predictor of poor outcome [14-20] and greater hospital expenses [21]. Moreover, two recent studies have shown that anemia associated with CKD were independent risk factors for one year mortality among patients with HF [22,23]. One study included only patients with left ventricular systolic dysfunction [22], whereas patients with left ventricular diastolic dysfunction were also included in the other [23]. Independent associations between both CKD and anemia with increased risk of one-year mortality were found. In both studies, a 1% decrease in hematocrit was associated with a 2.5% increase in the 12 month risk of death.

The purpose of our study was to examine, among patients with HF, the combined association of CKD and anemia on adverse outcomes. To our knowledge, this is the first study using inhospital mortality and early readmission for this purpose.

## Methods

### Study design

This was a retrospective cohort study of patients having a diagnosis of heart failure hospitalized and discharged between January 1- December 31, 1999 from two Swiss university hospitals. All adult patients with heart failure hospitalized in all wards for any reason were included in the study. Outcome measures of interest were inhospital mortality and 30-day readmissions. Follow-up for each patient began on the date of discharge from the hospital and continued for 30 days.

### Population

Using administrative data, we identified all patients hospitalized with a principal or secondary diagnosis of HF (International Classification of Disease, 10^th ^revision: I50.0, I50.1, I50.9, I11.0, I13.0 and I13.2 (ICD-10)). We then selected a random sample of 700 patients hospitalized in each hospital with HF among respectively 976 and 774. Patients were excluded from the sample if the initial hospitalization was terminated against medical advice, or if they were transferred to another acute care hospital or if no information on the creatinine level was available. Additional exclusion criteria included diagnosis of valvular heart disease, acute myocardial infarction, cor pulmonale, chronic obstructive pulmonary disease treated with home oxygen, thiamine deficiency, amyloidosis and thyrotoxicosis.

### Data

Data were abstracted from medical charts by medical record specialists. The entire medical chart was available in one hospital and the scanned medical record was used in the other. Variables abstracted from the chart included age, sex, smoking status, recorded history of previous heart failure, myocardial infarction, chronic obstructive pulmonary disease, bronchitis, emphysema, hypertension and diabetes. Clinical information included a history of paroxysmal nocturnal dyspnea, dyspnea on exertion (DOE) and orthopnea. Physical findings abstracted included pedal edema, pulmonary rales, S3-gallop and evidence of elevated jugular vein pressure. The presence of atrial fibrillation on the admission electrocardiogram (ECG) was recorded. Hemoglobin levels were distributed in four groups: <10 g/dL, 10 g/dL to 12 g/dL, 12 g/dL to 14 g/dL, and ≥ 14 g/dL. The final serum creatinine values recorded during the hospitalization were also considered. Chronic kidney disease (CKD) was defined as a serum creatinine ≥ 124 μmol/L for women and ≥ 133 μmol/L for men. We choose these ranges because they were used previously in an US study, in order to be able to do comparisons of CKD prevalence between countries [23]. We did not calculate creatinine clearance because, in many patients, the information available in our data set did not allow us to calculate it. A random replicate sample of 100 charts was abstracted to assess inter-rater reliability. The Kappa estimate was 0.91 for the determination of the ventricular function (VF) and 1.0 for inhospital mortality.

Information on inhospital mortality and readmission within 30 days was gathered using administrative data provided by the hospitals. We assessed all cause readmission and included only patients from the index hospital. Because these hospitals are university referral centers, each for a different area, we assumed that only few patients could have been readmitted to a different hospital. Indeed, for one provider, we could assess that none of the patients were readmitted to another Swiss hospital using a unique identifier from the Swiss Federal Statistical Office.

The determination of the left ventricular function was based on the chart by the presence of a value for a previously measured ejection fraction on echocardiography, cardiac catheterization, radionuclide ventriculography or by a narrative statement in the chart. Patients with left ventricular systolic dysfunction (LVSD) were identified by looking in medical charts for a current (from the index hospitalization) or previous ejection fraction (EF) equal or less than 40%. If no information regarding the EF was found, we searched for a narrative description in the chart. Specifically, the following terms were associated to LVSD: "systolic dysfunction." "dilated cardiomyopathy," "congestive cardiomyopathy," "diffuse global hypokinesis" or "systolo-diastolic dysfunction" (patients reported to have both systolic and diastolic dysfunction by cardiologists). Further, angiotensin converting enzyme inhibitor (ACEI) were identified in the medical charts through generic or trade name, including benazapril, captopril, enalapril, fosinopril, lisinopril, quinalapril, ramipril, perinopril and cilazapril.

The Charlson co-morbidity index, a weighted average of selected co-morbidities, was computed at index hospitalization for each patient as a measure of severity of illness measure using the Deyo modification [24].

### Statistical analysis

Bivariate analyses of the dependent and the primary exposure variables were conducted. We also calculated the crude risk ratio and 95% confidence intervals for inhospital mortality and 30-day readmission. We used chi-square tests, Fisher's exact tests, Student T-tests or ANOVA methods when appropriate. Dichotomous outcome variables were inhospital mortality and readmission within 30 days. Primary exposure variables were hemoglobin and creatinine levels. Other variables, potential confounding factors, included in the bivariate analysis were: hospital, age, sex, history of heart failure, diabetes mellitus, hypertension, prior myocardial infarction, chronic obstructive pulmonary disease, smoking, symptoms and findings at admission (paroxysmal nocturnal dyspnea, dyspnea on exertion, orthopnea, leg oedema, pulmonary rales, jugular vein distension, S3-gallop), atrial fibrillation, left ventricular function, ejection fraction, ACEI prescription at discharge, Charlson co-morbidity index, as well as inhospital length of stay.

We then performed multivariate analyses using logistic regression to adjust for potential confounding factors. Logistic regression was used to calculate adjusted odds ratio with associated 95% confidence intervals. Covariates were initially selected using a priori considerations as well as strength of association and statistical significance in bivariate analyses. We included the variable "Left ventricular function" in the starting model in order to control for the heterogeneity of the study population between diastolic and systolic HF. We first looked if interaction between hemoglobin and creatinine was significant. After defining the starting model as above, we assessed, by backward elimination, which confounding factors should remain in the model. We first looked to see if the least significant variable was a confounding factor by dropping it and refitting the model. We then assessed if the odds ratio changed by more than 10% compared to odds ratio of the starting model. If the odds ratios changed by more then 10%, the variable was considered as a potential confounding factor and remained in what became the final model. If a variable did not meet these criteria, it was removed from the model and the same procedures were reapplied until the best final model was found. Fit of the models was assessed using the Hosmer-Lemeshow goodness of fit test. For all models, we checked for any potential collinearity problems between the variables. All analyses were implemented with the SAS software, version 8.02 (SAS Institute Inc. Cary, NC, USA).

## Results

### Baseline characteristics

Our sample included 955 eligible patients with HF available for analysis. Among those 411 (43.0%) were admitted to hospital A and 544 (57.0%) in hospital B. The mean (SD) age was 75.4 years (12.8), 45.7% were female. A history of HF was present in 58.7% of the patients. A history of myocardial infarction was reported for 34.1%, hypertension for 60.7%, diabetes for 23.2%, and COPD or bronchitis or emphysema for 19.9%. At discharge, anticoagulants were prescribed in 28.7% of the patients, beta-blockers in 12.8%, calcium blockers in 13.3%, digoxin in 32.2%, diuretics in 59.9%, nitrates in 30.3%, angiotensin receptor blockers in 8.1% and spironolactone in 11.1%.

In our sample, based on a value of left ventricular ejection fraction or a narrative statement, 28.9% had their left ventricular function not determined, 28.0% had a left ventricular systolic dysfunction (LVSD), and 43.1% a left ventricular diastolic dysfunction. Further, a report describing previous or current value of left ventricular ejection fraction was found in 46.7% of the patient's charts. The mean (SD) ejection fraction was 36.0% (15.0%) with a 25^th ^to 75^th ^intraquartile range from 25 to 45%. An ACEI was prescribed at discharge in 61.2% of the patients. The mean (SD) Charlson comorbidity index was 2.2 (1.4). The median length of stay was 10 days, with a 25^th ^to 75^th ^intraquartile range from 6 to 17 days.

### Prevalence of CKD

The mean (SD) value of the last serum creatinine value reported during the hospitalization was 113.9 (54.0) μmol/L, with a range from 32 to 545 μmol/L and a 25^th ^to 75^th ^intraquartile range from 84 to 126 μmol/L. Chronic kidney disease was defined as a serum creatinine ≥ 124 μmol/L in women and ≥ 133 μmol/L in men. Men (25.3%) were more likely than women (20.4%) to have CKD. In total, 220 (23.0%) patients of the entire cohort had CKD. The mean serum creatinine value was statistically significantly higher in patients with a history of myocardial infarction, hypertension, diabetes or leg edema (Table 1). Higher creatinine values were also observed in patients with a Charlson comorbidity index larger than 2 (Table 2).

**Table 1:** Patients Characteristics at Admission in Patients with Heart Failure and Proportion with Chronic Kidney Disease, N = 955

**Characteristics**	N (%)	Chronic Kidney Disease (%) * N (%)	**Mean (SD) serum creatinine μmol/l**
N (%)	955 (100.0)	220 (23.0)	113.9 (54.0)
Hospital A	411 (43.0)	109 (26.5)	116.4 (56.2)
Hospital B	544 (57.0)	111 (20.4)**	112.0 (52.3)
Age (N = 955)			
16 – 60 years	114 (11.9)	23 (20.2)	111.4 (56.2)
61 – 70 years	172 (18.0)	33 (19.2)	110.2 (50.7)
71 – 80 years	282 (29.5)	61 (21.6)	113.2 (56.0)
> 80 years	387 (40.5)	103 (26.6)	116.7 (53.5)
Sex (N = 954)			
Male	518 (54.3)	131 (25.3)	120.1 (55.4)
Female	436 (45.7)	89 (20.4)	106.5 (51.5)**
Previous history Heart Failure (N = 876)	514 (58.7)	126 (24.5)	115.6 (53.4)
Prior MI (N = 945)	322 (34.1)	84 (26.1)	119.2 (50.7)**
COPD, bronchitis, Emphysema (N = 944)	188 (19.9)	37 (19.7)	107.3 (45.8)**
Hypertension (N = 950)	577 (60.7)	144 (25.0)	117.8 (57.7)**
Diabetes (N = 952)	221 (23.2)	57 (25.8)	122.3 (58.4)**
Current smoker (N = 930)	141 (15.2)	25 (17.7)	104.7 (43.0)**
Symptoms and findings			
PND (N = 608)	163 (26.8)	37 (22.7)	114.1 (50.1)
DOE (N = 849)	672 (79.2)	158 (23.5)	113.8 (50.9)
Orthopnea (N = 655)	330 (50.4)	92 (27.9)**	111.1 (56.4)
Leg edema (N = 791)	438 (55.4)	117 (26.7)**	118.0 (59.0)**
Pulmonary rales (N = 815)	508 (62.3)	124 (24.4)	115.0 (53.9)
S3 gallop (N = 757)	39 (5.2)	8 (20.5)	111.4 (32.6)
JVD (N = 700)	236 (33.7)	66 (28.0)**	118.2 (62.0)
Atrial fibrillation (N = 797)	221 (27.7)	54 (24.4)	110.0 (38.9)

MI: Myocardial Infarction; COPD: Chronic Obstructive Pulmonary Disease; PND: Paroxystal Nocturnal Dyspnea; DOE: Dyspnea On Exertion; JVD: Jugular Vein Distension

* Chronic kidney disease (CKD) was defined as a serum creatinine > = 124 μmol/L for women and > = 133 μmol/L for men

** p value < 0.05

**Table 2:** Hospital Characteristics in Patients with Heart Failure and Proportion with Chronic Kidney Disease, N = 955

**Characteristics**	N (%)	Chronic Kidney Disease (%) * N (%)	**Mean (SD) serum creatinine μmol/l**
N (%)	955 (100.0)	220 (23.0)	113.9 (54.0)
			
Ejection fraction in % (N = 446)			
< = 20	77 (17.3)	20 (26.0)	121.3 (45.1)
20–30	128 (28.7)	29 (22.7)	115.9 (65.2)
30–40	108 (24.2)	28 (25.9)	116.8 (49.2)
> 40	133 (29.8)	37 (27.8)	120.2 (63.5)
			
Left ventricular function (N = 955)			
Diastolic dysfunction	412 (43.1)	98 (23.8)	115.7 (51.1)
Systolic dysfunction	267 (28.0)	61 (22.9)	116.6 (64.6)
Undetermined	276 (28.9)	61 (22.1)	108.5 (46.3)
			
Discharged with ACEI (N = 884)	541 (61.2)	109 (20.2)	108.1 (36.2)**
			
Charlson comorbidity index (N = 955)			
0–1	398 (41.7)	55 (13.8)	102.4 (38.5)
2	216 (22.6)	28 (13.0)	102.2 (40.0)
>2	341 (35.7)	137 (40.2)**	134.6 (69.1)**
			
Inhospital mortality (N = 955)	89 (9.3)	44 (49.4)**	159.3 (106.1)**
30 days readmissions (N = 866)	116 (13.4)	26 (22.4)	107.7 (38.7)
Length of stay (days) (N = 955)			
0–6	247 (25.9)	58 (23.5)	118.3 (55.1)
7–12	321 (33.6)	69 (21.5)	112.6 (53.6)
>12	387 (40.5)	93 (24.0)	112.0 (53.7)

ACEI: Angiotensin Converting Enzyme Inhibitors

* Chronic kidney disease (CKD) was defined as a serum creatinine > = 124 μmol/L for women and > = 133 μmol/L for men

** p value < 0.05

### Prevalence of anemia

Hemoglobin level was recorded for 920 members (96%) of the cohort. The mean (SD) hemoglobin was 13.0 g/dL (2.2) with a 25^th ^to 75^th ^intraquartile range from 11.8 g/dL to 14.6 g/dL. On admission, an hemoglobin of ≥ 14 g/dL was found in 36.1% of the patients, 36.3% had an hemoglobin between 12 g/dL and 14 g/dL, 19.6% between 10 g/dL and 12 g/dL, and 8% ≤ 10 g/dL.

The proportion of patients with CKD was associated with increasing anemia (Table 3). The mean serum creatinine was increasing with severity of anemia (Table 3) from 102.0 μmol/L among patients with no anemia, up to 141.0 μmol/L for severe anemia (p < 0.0001). Patients with severe anemia were more likely not to be discharged with ACEI. (Table 3).

**Table 3:** Hospital Characteristics in Patients with Heart Failure According to Hemoglobin Level, N = 955

	**Hemoglobin in g/dL**			
	**<10 N (%) or Mean (SD)**	**10–12 N (%) or Mean (SD)**	**12–14 N (%) or Mean (SD)**	**≥14 N (%) or Mean (SD)**

N (%) (N = 920)	74 (8.0)	180 (19.6)	334 (36.3)	332 (36.1)
CKD (N = 920)*	28 (37.8)	61 (33.9)	78 (23.4)	48 (14.5)
Mean (SD) serum creatinine (N = 920)* (μmol/L)	141.0 (91.0)	131.6 (76.5)	110.3 (42.5)	102.0 (30.7)
Left ventricular function (N = 920)*				
Diastolic dysfunction	19 (25.7)	70 (38.9)	136 (40.7)	171 (51.5)
Systolic dysfunction	29 (39.2)	58 (32.2)	89 (26.7)	80 (24.1)
Undetermined	26 (35.1)	52 (28.9)	109 (32.6)	81 (24.4)
Mean (SD) ejection fraction in % (N = 429)*	41.8 (14.7)	39.7 (14.7)	35.9 (15.5)	34.1 (14.3)
Discharged with ACEI (N = 854)*	31 (45.6)	108 (63.2)	196 (63.2)	193 (63.2)
Hospital A (N = 406)	27 (6.7)	72 (17.7)	153 (37.7)	154 (37.9)
Hospital B (N = 514)	47 (9.1)	108 (21.0)	181 (35.2)	178 (34.6)
Inhospital mortality (N = 920)*	14 (18.9)	18 (10.0)	31 (9.3)	18 (5.4)
30 days readmissions (N = 839)	8 (13.3)	29 (17.9)	38 (12.5)	36 (11.5)
Mean (SD) length of stay (days) (N = 920)*	18.8 (21.3)	16.8 (16.4)	13.8 (17.0)	12.4 (9.5)

CKD: Chronic Kidney Disease; ACEI: Angiotensin Converting Enzyme Inhibitors

*p value < 0.05

### Mortality and readmission

Eighty-nine (9.3%) patients died during their hospitalization, 20% among those with CKD and 6.1% among those without CKD (p < 0.0001). Among patients who died in the hospital, 49.4% had CKD, and their mean (SD) serum creatinine value was 159.3 μmol/L (106.1) (p < 0.0001).

Anemia on admission to the hospital was associated with increased risk of death. In-hospital mortality was 5.4% for patients with a hemoglobin of ≥ 14 g/dL, 9.3% for a hemoglobin between 12 g/dL and 14 g/dL, 10.0% for a hemoglobin between 10 g/dL and 12 g/dL, and 18.9% for a hemoglobin < 10 g/dL (p = 0.002) (Table 3). In-hospital mortality rates were also higher in patients with COPD (Table 4) and in patients with a Charlson comorbidity index over 2. Individuals with left ventricular diastolic and systolic dysfunction, as well as those with undetermined ventricular function, had comparable risk of hospital death (Table 5).

**Table 4:** Patients Characteristics at Admission in Patients Hospitalized with Heart Failure by Outcome Indicators, N = 955

	**Inhospital mortality N = 955**		**30 day readmission N = 866**	
**Patients Characteristics**	**N**	**N dead (%) 89 (9.3%)**	**N**	**N Readmitted (%) 116 (13.4%)**

Hospital A	411	41 (10.0)	370	43 (11.6)
Hospital B	544	48 (8.8)	496	73 (14.7)
Age (N = 955)				
16 – 60 years	114	7 (6.1)	107	18 (16.8)
61 – 70 years	172	12 (7.0)	160	20 (12.5)
71 – 80 years	282	23 (8.2)	259	43 (16.6)
> 80 years	387	47 (12.1)	340	35 (10.3)
Sex (N = 954)				
Male	518	49 (9.5)	469	64 (13.7)
Female	436	40 (9.2)	396	52 (13.1)
Previous history HF (N = 876)	514	50 (9.7)	464	51 (11.0)*
Prior MI (N = 945)	322	29 (9.0)	293	35 (12.0)
COPD, bronchitis, emphysema (N = 944)	188	26 (13.8)*	162	32 (19.8)*
Hypertension (N = 950)	577	49 (8.5)	528	68 (12.9)
Diabetes (N = 952)	221	20 8 (9.0)	201	34 (16.9)
Current smoker (N = 930)	141	13 (9.2)	128	24 (18.8)*
Symptoms and findings				
PND (N = 608)	163	9 (5.5)	154	24 8 (15.6)
DOE (N = 849)	672	49 (7.3)	623	80 (12.8)
Orthopnea (N = 655)	330	22 (6.7)	308	43 (14.0)
Leg edema (N = 791)	438	39 (8.9)	399	55 (13.8)
Pulmonary rales (N = 815)	508	35 (6.9)	473	53 (11.2)
S3 gallop (N = 757)	39	2 (5.1)	37	3 (8.1)
JVD (N = 700)	236	20 (8.5)	216	31 (14.4)
Atrial fibrillation (N = 797)	221	9 (4.1)	212	24 (11.3)

HF : Heart Failure; MI: Myocardial Infarction; COPD: Chronic Obstructive Pulmonary Disease; PND: Paroxystal Nocturnal Dyspnea; DOE: Dyspnea On Exertion; JVD: Jugular Venous Distension

*p value < 0.05

**Table 5:** Hospital Characteristics in Patients with Heart Failure by Outcome Indicators, N = 955

	**Inhospital mortality N = 955**		**30 day readmission N = 866**	
**Patients Characteristics**	**N**	**N dead (%) 89 (9.3%)**	**N**	**N Readmitted (%) 116 (13.4%)**

Ejection fraction in % (N = 446)				
< = 20	77	8 (10.4)	69	7 (10.1)
20–30	128	13 (10.2)	115	20 (17.4)
30–40	108	5 (4.6)	103	12 (11.7)
> 40	133	14 (10.5)	119	23 (19.3)
				
Left ventricular function (N = 955)				
Diastolic dysfunction	412	35 (8.5)	377	54 (14.3)
Systolic dysfunction	267	24 (9.0)	243	37 (15.2)
Undetermined	276	30 (10.9)	246	25 (10.2)
				
Discharged with ACEI (N = 836)	NA	NA	532	76 (14.3)
				
Charlson comorbidity index (N = 955)				
0–1	398	26 (6.5)	372	47 (1.6)
2	216	20 (9.3)	196	24 (12.2)
>2	341	43 (12.6)*	298	45 (15.1)
				
Length of stay (days) (N = 955)				
0–6	247	32 (13.0)	215	26 (12.1)
7–12	321	17 (5.3)	304	36 (11.8)
>12	387	40 (10.3)*	347	54 (15.6)

ACEI: Angiotensin Converting Enzyme Inhibitor; CHF: Congestive Heart Failure; NA: not applicable because relates only to patients discharged alive.

*p value < 0.05

Among 866 patients discharged alive, 116 (13.4%) were readmitted within 30 day, 14.0% of patients with CKD, and 12.9% of those without CKD. Early readmission occurred in 11.5% of patients with a hemoglobin of ≥ 14 g/dL, 12.5% for a hemoglobin between 12 g/dL and 14 g/dL, 17.9% for a hemoglobin between 10 g/dL and 12 g/dL and 13.3% for a hemoglobin < 10 g/dL. Patients who were current smokers and with COPD were also more likely to be readmitted (Table 4).

### Multivariate analysis

Both hemoglobin and serum creatinine were independently associated with poor outcomes after controlling for confounding factors (Table 6). For inhospital mortality, the model controlled for length of stay and COPD. For each g/dL increase in hemoglobin, the inhospital mortality rate declined by 39% (p = 0.0008). For each one μmol/L increase in serum creatinine, inhospital mortality rate decreased by 1% (p = 0.166). Further, the interaction term between hemoglobin and serum creatinine was statistically significant (p = 0.008). At the mean creatinine level, increasing hemoglobin levels were associated with lower mortality (RR = 0.86, for each unit increase in hemoglobin). Effect modification, suggested a weaker association of hemoglobin with mortality as creatinine levels increased. Further, at the mean level hemoglobin, increasing creatinine levels were associated with higher mortality (RR = 1.015, for each unit increase in creatinine).

**Table 6:** Results of the Logistic Regression Models, N = 910

	**Parameter**	**Standard Error**	**P Value**
**Risk Factors**			
**Model for Inhospital Mortality, N = 910**			
Hemoglobin in g/dL	-0.3898	0.1159	0.0008
Serum creatinine in μmol/L	-0.0122	0.0088	0.166
Hemoglobin in g/dL × Serum creatinine in μmol/L	0.0021	0.0008	0.008
Length of stay	0.0063	0.0060	0.296
COPD	0.8079	0.2846	0.005
			
**Model for 30 Day Readmission, N = 767**			
Hemoglobin in g/dL	-0.1300	0.0499	0.009
Serum creatinine in μmol/L	-0.0008	0.0026	0.744
Hemoglobin in g/dL × Serum creatinine in μmol/L	NA	NA	NA
Age	-0.0129	0.0079	0.101
History of heart failure	-0.4308	0.2187	0.049
COPD	0.6666	0.2536	0.009

COPD: Chronic Obstructive Pulmonary Disease; NA: not applicable because not statistically significant and therefore not in the final best model

In the multivariate analysis using 30 days readmission as dependent variable, we controlled for age, COPD and history of heart failure. The interaction term between hemoglobin and serum creatinine was not statistically significant. Results showed that for each one g/dL increase in hemoglobin, readmission rate declined by 13% (p = 0.009). Further, for each one μmol/L increase in serum creatinine, readmission rate increased by 0.08% (p = 0.744).

After controlling for all other risk factors, the odds ratio related to inhospital mortality associated with the presence of anemia defined as hemoglobin less than 12 g/dL, was 1.47 (95% CI 0.89 to 2.42) in all heart failure patients and 4.04 (95% CI 2.46 to 6.66) in patients with additional CKD compared with HF patients who had a hemoglobin level ≥ 12 g/dL and no CKD. Similarly, the odds ratio for early readmissions were 1.60 (95% CI 1.00 to 2.58) for anemia and 1.14 (95% CI 0.67 to 1.93) for CKD. In these models, the interaction terms between anemia and CKD lacked statistical significance.

## Discussion

In this study, both anemia and chronic kidney disease were highly prevalent among HF patients discharged from two university hospitals and independently associated with an increased risk of dying in the hospital or of being readmitted within 30 days. The association between CKD, anemia and these outcomes (inhospital mortality and readmission) in HF patients has not been reported previously. Most studies have focused only on survival after hospital discharge as an outcome.

One study, in the framework of the SOLVD study, included only patients with left ventricular dysfunction. The risk of increased mortality associated with a 1% reduction in hematocrit was 2.7% [22]. These results were comparable to another study conducted among Medicare beneficiaries in community hospitals in the US. In this latest study, patients with left ventricular diastolic dysfunction were also included as patients with left ventricular systolic dysfunction. The risk of death associated with a 1% reduction in hematocrit was 2% [23]. In a new large recent study, among HF patients, chronic kidney disease and anemia were found independently to confer a twofold increased risk of death [25]. Silverberg et al. recently reported that, in a randomized trial of 32 ambulatory HF patients with NYHA class III and IV and an hemoglobin < 12 g/dL, the correction of anemia was associated with an improved functional status and decreased hospitalization. However, the major limitation of this study was its small sample size and the fact that the randomization was not blinded [26]. These observations suggest that anemia is a clinically important risk factor for death and readmission among heart failure patients, with or without CKD. The clinical implication of these findings for patients with HF is that failure to correct severe anemia among patients with CKD confers a preventable burden of reduced quality of life, while clinical trials have demonstrated that correction of anemia improved these measures. HF patients should be carefully examinated for presence of CKD and anemia and, if present, treated according to current evidence [27]. Treating anemia among inpatients with HF and CKD may then reduce inhospital mortality and early readmission. However, currently no large clinical trials have been conducted to evaluate the effect of erythropoietin therapy on survival or readmission among patients suffering from HF and CKD.

In our study we found that, among HF patients, the prevalence of CKD was 25% among males and 20% among females respectively. However, by using this cut-point of a serum creatinine of ≥ 124 μmol/L for women and ≥ 133 μmol/L for men for defining CKD, we underestimated the true prevalence of CKD especially among elderly people. We choose these cut-points based on previous studies implemented in the USA [23]. Reduced kidney function occurred frequently in patients with HF. Two studies have shown that creatinine clearance less than 60 ml/minute was present in 20 to 50% of HF patients [28,29]. In another study, which included Medicare beneficiaries with heart failure hospitalized in community hospitals, 38% had CKD. In this cohort, the prevalence of CKD was 33% in females and 46% in males [23]. Our results are similar to those found in these studies and show that CKD is highly prevalent among HF patients.

Interest in the relationship between HF and anemia is growing. Anemia commonly complicates HF (14–28% of patients depending on the cut off used) [30] and is a potential exacerbating factor [31]. In our study the prevalence of anemia (hemoglobin < 12 g/dL) among HF patients was 28%. In another study performed in one Swiss university hospital, the prevalence of anemia was 15% [13]. Silverberg et al. showed that the prevalence of anemia increased with the severity of HF and reached almost 80% in those patients with a NYHA class IV [26]. Anemia observed among individuals with HF is highly multi-factorial, but, a decreased renal function is a cause in numerous patients [32].

Anemic patients with chronic renal failure should receive treatment with recombinant human erythropoietin (r-HuEPO, Eéoetin) to maintain hemoglobin levels over 11 g/dL with an acceptable target of 12 to 12.5 g/dL, according to recommendations from the European practice guideline for management of anemia in patients with chronic renal failure [33] and the National Kidney Foundation K/DOQI clinical practice guidelines for anemia of chronic kidney disease [27]. Benefits of adequate hemoglobin levels had been established in patients undergoing dialysis, and are supposed to be relevant also in CKD patients. In addition, anemic patients should receive iron supplementation in order to maintain serum ferritin levels above 100 μg/L and transferrin saturation above 20%.

This study had a number of limitations. It is an observational study based on information available in medical records. The chart abstraction process was implemented in each hospital by different persons with different education and backgrounds, although with similar training. Then, in one hospital the entire medical chart was available to the abstractors, whereas in the other only the electronic discharge letter, laboratory findings and reports from cardiology testing were available. In addition, the quality of medical records and completeness of information may also vary between centers. Incorrect information may have led to some misclassification bias. Further this study was conducted on an opportunity sample of two hospitals, making the generalisibility of results uncertain. Then, we excluded patients with valvular heart disease and acute myocardial infarction, because it was our intent to focus on a homogenous group of individuals with established heart failure. However, we agree that the issue of anemia and outcomes in both of these patient groups is important. Further, we were not able to exclude other causes of anemia, including the presence of iron, folate and vitamin B12 deficiencies, dilutional anemia, and the anemia of chronic diseases different from CKD, as explanations for anemia observed in this population and perhaps, to account for some potential confounders. Then, given the relative high number of elderly patients in the study population and that these patients may have CKD even with normal creatinine value; we underestimated the true prevalence of CKD. Finally, we acknowledge that we were not able to measure others risk factors associated with epo-resistance such as immune activation. We will consider measuring it in future studies. However, we would like to emphasis that the concerns about ACEI and anemia should not keep physicians from using ACE inhibitors in their management of heart failure.

## Conclusion

In conclusion, we found further evidence that the concomitant presence of either CKD or anemia increased the risk of dying in the hospital or of being readmitted within 30 days among patients hospitalized with heart failure. The association persisted after controlling for other factors associated with adverse outcomes in these patients.

## Competing interests

This study was supported by a grant from the coalition of the five Swiss University Hospitals and grants from the Fonds du 450ème anniversaire de l'Université et la Fondation Moffat. It was also sponsored by MSD Switzerland and Roche Switzerland. These sponsors were however not involved in the analysis of the results neither in writing nor in correcting the manuscript.

## Authors' contributions

JCL participated in the conception and design of the study, acquisition of data, analysis and interpretation of data, as well as drafting the manuscript. WDF participated in the design of the study, supervised the statistical analysis, and revised critically the article. MB and BB participated in the conception and design of the study, interpretation of data and revised critically the manuscript. WMM conceived of the study, participated in its design, interpretation of data and revised critically the article. All authors read and approved the final manuscript.

## Pre-publication history

The pre-publication history for this paper can be accessed here:


